# Iminosugar-Based Inhibitors of Glucosylceramide Synthase Increase Brain Glycosphingolipids and Survival in a Mouse Model of Sandhoff Disease

**DOI:** 10.1371/journal.pone.0021758

**Published:** 2011-06-29

**Authors:** Karen M. Ashe, Dinesh Bangari, Lingyun Li, Mario A. Cabrera-Salazar, Scott D. Bercury, Jennifer B. Nietupski, Christopher G. F. Cooper, Johannes M. F. G. Aerts, Edward R. Lee, Diane P. Copeland, Seng H. Cheng, Ronald K. Scheule, John Marshall

**Affiliations:** 1 Genzyme Corporation, Framingham, Massachusetts, United States of America; 2 Academic Medical Center, University of Amsterdam, Amsterdam, The Netherlands; Baylor Research Institute, United States of America

## Abstract

The neuropathic glycosphingolipidoses are a subgroup of lysosomal storage disorders for which there are no effective therapies. A potential approach is substrate reduction therapy using inhibitors of glucosylceramide synthase (GCS) to decrease the synthesis of glucosylceramide and related glycosphingolipids that accumulate in the lysosomes. Genz-529468, a blood-brain barrier-permeant iminosugar-based GCS inhibitor, was used to evaluate this concept in a mouse model of Sandhoff disease, which accumulates the glycosphingolipid GM2 in the visceral organs and CNS. As expected, oral administration of the drug inhibited hepatic GM2 accumulation. Paradoxically, in the brain, treatment resulted in a slight increase in GM2 levels and a 20-fold increase in glucosylceramide levels. The increase in brain glucosylceramide levels might be due to concurrent inhibition of the non-lysosomal glucosylceramidase, *Gba2*. Similar results were observed with *N*B-DNJ, another iminosugar-based GCS inhibitor. Despite these unanticipated increases in glycosphingolipids in the CNS, treatment nevertheless delayed the loss of motor function and coordination and extended the lifespan of the Sandhoff mice. These results suggest that the CNS benefits observed in the Sandhoff mice might not necessarily be due to substrate reduction therapy but rather to off-target effects.

## Introduction

Sandhoff disease, or type 2 GM2 gangliosidosis, is caused by mutations in the *HEXB* gene and the resultant deficiency in β-hexosaminidase activity. This deficiency causes aberrant lysosomal accumulation of the ganglioside GM2, β-*N*-acetylgalactosamine-terminal glycolipids and β-*N*-acetylglucosamine-terminal oligosaccharides [1]. Sandhoff disease manifests primarily as a neuropathic disease of infants, though subjects exhibit a range of severities, as noted for most lysosomal storage disorders. Presently, there are no approved therapies for Sandhoff disease.

Glycosphingolipidoses that do not present with neuropathic disease (such as type 1 Gaucher disease and Fabry disease) can be treated effectively by enzyme-replacement therapy (ERT) by periodic intravenous infusions of the respective recombinant enzymes [2], [3]. However, ERT does not address the CNS manifestations of neuropathic glycosphingolipidoses (e.g. types 2 and 3 Gaucher disease, GM1 and GM2 gangliosidoses) since the lysosomal enzymes are unable to traverse the blood-brain barrier [4], [5]. An alternative therapeutic strategy is to target glucosylceramide synthase (GCS), the enzyme that catalyzes the first step in the biosynthesis of glycosphingolipids. Inhibition of GCS effects what is commonly referred to as substrate reduction therapy (SRT). SRT is designed to abate the synthesis of glucosylceramide, and by extension the aberrant lysosomal storage of glycosphingolipids. Indeed, preclinical and clinical studies in type 1 Gaucher disease have shown that such inhibitors significantly improve the disease manifestations in the viscera [6], [7]. Preclinical studies with a GCS inhibitor have also shown that SRT is potentially therapeutic in Fabry disease [8].

Presently, one GCS inhibitor (miglustat) has been approved for use in mild to moderate type 1 Gaucher patients for whom ERT is not a therapeutic option and in Niemann-Pick C patients [6], [9]. Another, eliglustat tartrate, is in phase 3 clinical trials for type 1 Gaucher patients [7]. Miglustat but not eliglustat tartrate is able to traverse the blood-brain barrier and thus might be used to treat the neuropathic glycosphingolipidoses. While excessive inhibition of the glycosphingolipid biosynthetic pathway could be detrimental to neuronal development and stabilization [10]–[12], the goal is to effect a partial reduction such that the rate of synthesis is matched by the residual capacity of the cells to degrade the substrates. Accordingly, SRT is arguably best suited for those indications that retain some measure of residual enzyme activity as in type 3 Gaucher patients and late-onset Tay-Sachs diseases.

As iminosugar-based GCS inhibitors (*N*-butyl-deoxynojirimycin (*N*B-DNJ), or miglustat) have been shown capable of entering the brain parenchyma and improving disease outcomes in mouse models of lysosomal storage disorders [13]–[15], we have elected to evaluate structural analogs that are reportedly more potent. One such molecule is AMP-DMP or Genz-529468 [16], [17] as it is referred to in this report. Testing was performed in a mouse model of Sandhoff disease [18] that lacks β-hexosaminidase activity and accumulates GM2 and GA2 throughout the CNS, liver and kidney. CNS manifestations are apparent by 3 months of age and progressive, with death occurring at 4–5 months of age. The CNS involvement is the likely cause of death, as the peripheral nervous system shows no significant abnormalities [19]. The potential utility of SRT in Sandhoff disease was elegantly demonstrated by the generation of a mouse that was deficient in both β-hexosaminidase and GM2/GA2 synthase [20]. The resulting double knockout mouse no longer accumulated glycosphingolipids and exhibited an increased lifespan.

Previous studies have shown survival benefit when Sandhoff mice were treated with the GCS inhibitor *N*B-DNJ or its galactose analog [13], [21], [22]. As Genz-529468 is approximately 250-fold more potent as a GCS inhibitor than *N*B-DNJ [23], we sought to evaluate its therapeutic potential for neuropathic glycosphingolipidoses. Our studies showed that Genz-529468 was comparable to *N*B-DNJ at increasing the survival of Sandhoff mice, but could do so at much lower doses. However, notable treatment-induced increases in CNS glycosphingolipids, particularly of glucosylceramide, suggest that the mechanism of action of the iminosugar GCS inhibitors is not necessarily through the anticipated mechanism of substrate reduction.

## Results

### Iminosugar-based glucosylceramide synthase inhibitors reduce GM2 levels in the livers of Sandhoff mice

To test the inhibitory activities of the GCS inhibitors, Genz-529468 and *N*B-DNJ, were administered to Sandhoff mice through their food (100 mg/kg/day and 600 mg/kg/day, respectively) starting at 25 days of age. As a comparator, eliglustat tartrate (Genz-112638), which is non blood-brain barrier permeant, was included in the study. Analysis of glycosphingolipid levels in the livers of drug-treated Sandhoff mice showed a 40–60% reduction in GM2 levels when compared to age-matched untreated Sandhoff mice at 112 days of age [data not shown and 21]. Hence, the formulations of Genz-529468 and *N*B-DNJ used in these studies were equally active at inhibiting non-CNS GCS despite using a 6-fold lower dose of Genz-529468.

### Genz-529468 and *N*B-DNJ significantly increase glucosylceramide levels in the brains of Sandhoff mice

**Figure 1:**
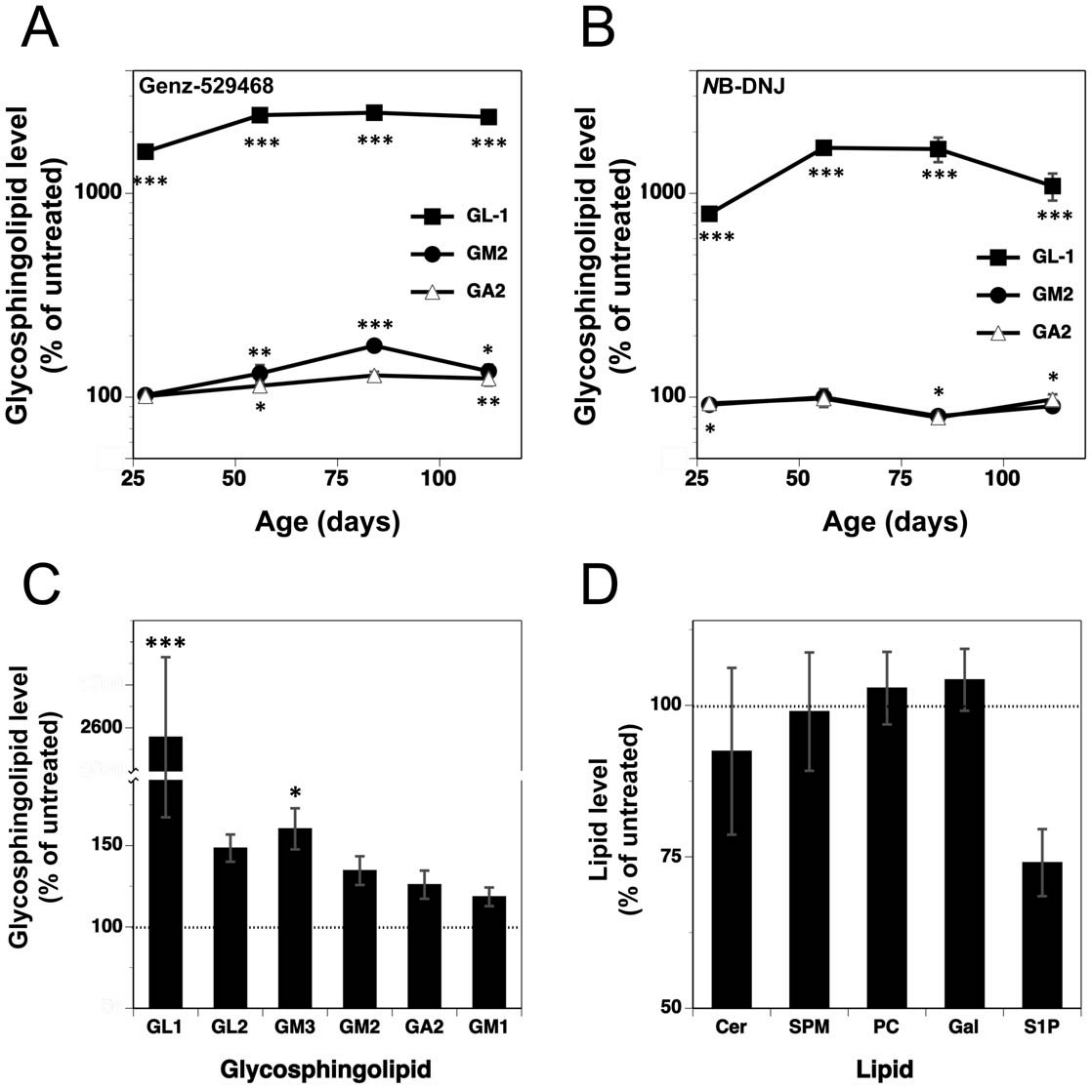
Iminosugar-based GCS inhibitors increase brain glycosphingolipid levels. Beginning at 25 days of age, Sandhoff mice were treated with either (**A**) Genz-529468 or (**B**) *N*B-DNJ, sacrificed over time and their brain tissue glycosphingolipids quantified (n = 4–5 mice per group per time point). Brain levels of GL1, GM2 and GA2 are shown relative to amounts in untreated age-matched controls. A larger range of brain (**C**) glycosphingolipids and (**D**) lipids, are shown following treatment with Genz-529468 at 112 days of age. Cer = ceramide, SPM = sphingomyelin, PC = phosphatidylcholine, Gal = galactosylceramide, S1P = sphingosine-1-phosphate. Statistics were performed between Sandhoff mice that were untreated and treated with drug, and were determined using the Graphpad Prism software t test; * = p<0.05, ** = p<0.01, *** = p<0.001. Error bars indicate SEM.

To evaluate whether the inhibitors (Genz-529468 and *N*B-DNJ) were also active in the CNS of Sandhoff mice, their brains were weighed and analyzed for changes in glycosphingolipid levels. Brain weight as a ratio to body weight was not significantly different between wild-type and Sandhoff mice at 112 days of age, and was unaffected by treatment with Genz-529468 or *N*B-DNJ (data not shown). Glycosphingolipid analysis was focused on the proximal target of the inhibitors, namely glucosylceramide (GL1), together with the two main storage products found in Sandhoff disease, the gangliosides GM2 and GA2. Untreated Sandhoff and wild-type mice harbor similar levels of GL1, but the former have >100-fold higher levels of GM2 and GA2 (data not shown). Contrary to our expectations, Sandhoff mice treated with Genz-529468 beginning at 25 days of age showed a rapid increase in brain GL1 levels, with levels rising to >10-fold those of untreated mice after 2–3 days of treatment (**Figure 1A**). These GL1 levels continued to increase further, rising to >20-fold those of untreated mice at all subsequent time points assayed (56, 84 and 112 days of age). Significant increases in GA2 and GM2 were also observed (**Figure 1A**), though these increases were more modest (120–150% those of untreated Sandhoff mice). Sandhoff mice treated with *N*B-DNJ resulted in a similar temporal profile of brain glycolipids, with GL1 levels increasing to >10-fold higher than those of untreated Sandhoff control mice (**Figure 1B**), However, in contrast to the effects of Genz-529468, *N*B-DNJ effected a modest but significant reduction in GM2 (∼90% of untreated mice) at 84 and 112 days of age, and had no effect on GA2 levels. Treating Sandhoff mice with the non-CNS permeant GCS inhibitor Genz-112638 (eliglustat tartrate) had no impact on brain glycosphingolipid levels.

In a separate study, Sandhoff mice were treated with Genz-529468 as in **Figure 1A**, and brain tissue at day 112 was analyzed for the levels of a panel of glycosphingolipids and other lipids. **Figure 1C** shows that in addition to the >20-fold increase in GL1 levels in the treated mice (as in **Figure 1A**), the relative levels of other glycosphingolipids in the pathway (GL2, GM3, GM2, GA2 and GM1) were also increased to 120–150% of untreated mice. Genz-529468 treatment had no effect on the relative brain levels of ceramide (Cer), sphingomyelin (SPM), phosphatidylcholine (PC) and galactosylceramide (Gal), but sphingosine-1-phosphate (S1P) levels were reduced to about 75% those of untreated Sandhoff mice (**Figure 1D**). Hence, enteric administration of the iminosugar-based GCS inhibitors Genz-529468 and *N*B-DNJ acted to increase rather than decrease glycosphingolipid levels in the brains of Sandhoff mice. In particular, GL1, the proximal glycosphingolipid in the GCS pathway, was increased many fold by both inhibitors. This effect on brain GL1 levels did not appear to be restricted to the Sandhoff mouse, as similar increases in brain GL1 levels were found in other mouse strains following treatment with Genz-529468 or *N*B-DNJ (data not shown).

### Treatment with iminosugar-based GCS inhibitors delays the development of pathology in Sandhoff mouse brain

**Figure 2:**
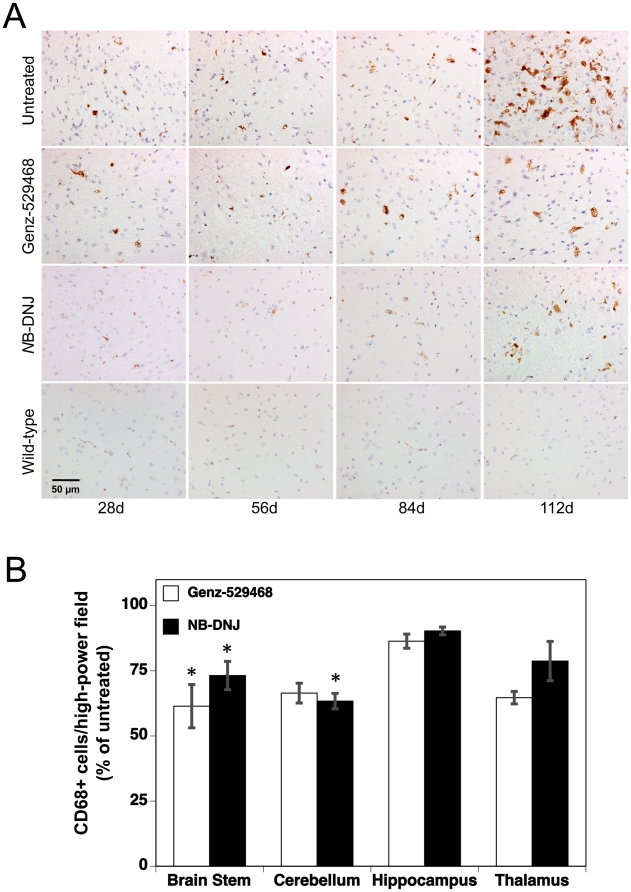
Iminosugar-based GCS inhibitors decrease the number of brain CD68 positive cells. (**A**) CD68 immunolabeling of brain stem from 28, 56, 84 and 112 day old Sandhoff mice, untreated or treated with either Genz-529468 or *N*B-DNJ. Dark brown cells are positive for CD68; scale bar = 50 µm. (**B**) Quantification of CD68^+^ cell counts in the brain stem, cerebellum, hippocampus and thalamus of 112 day old drug-treated Sandhoff mice (n = 4–5 mice per group). Cell counts are presented relative to those in untreated Sandhoff mice. Statistics are between untreated and treated Sandhoff mice, and were determined using the Graphpad Prism software t test; * = p<0.05. Error bars indicate SEM.

To evaluate the effects of the iminosugar-based GCS inhibitors on the brain pathology of Sandhoff mice, time-dependent changes in CD68 immunopositive cells, glial fibrillary acidic protein (GFAP) expression, and α-synuclein immunopositive cells were determined. CD68 is a marker for cells of the monocyte lineage (macrophages, dendritic cells, microglia), granulocytes and activated T cells. In wild-type mice, CD68^+^ cells were absent or rare within the neuroparenchyma, though a few scattered CD68^+^ cells were occasionally observed in leptomeninges. In contrast, Sandhoff mice exhibited significant numbers of CD68^+^ cells in the brain stem, cerebellum, hippocampus and thalamus. A few scattered CD68^+^ cells were also observed in the cerebral cortex and leptomeninges. **Figure 2A** shows the time-dependent changes in CD68^+^ staining in the brain stem of untreated wild-type and Sandhoff mice and Sandhoff mice treated with Genz-529468 or *N*B-DNJ. Untreated Sandhoff mice exhibited a higher number of CD68^+^ cells than age-matched wild-type mice at all ages. This increase was exacerbated as the Sandhoff mice aged, with a dramatic increase in CD68^+^ cells noted between 84 and 112 days of age. Treatment with either Genz-529468 or *N*B-DNJ appeared to reduce the number of CD68^+^ cells at each time point. This reduction was also apparent in other regions of the brain (**Figure 2B**). Quantitative analysis of the cerebellum, hippocampus and thalamus of Sandhoff mice treated with Genz-529468 or *N*B-DNJ at day 112 showed a similar decrease in the number of CD68^+^ cells noted in the brain stem. These observations are consistent with previous studies with GCS inhibitors [21], [24] or bone marrow transplantation [25].

**Figure 3:**
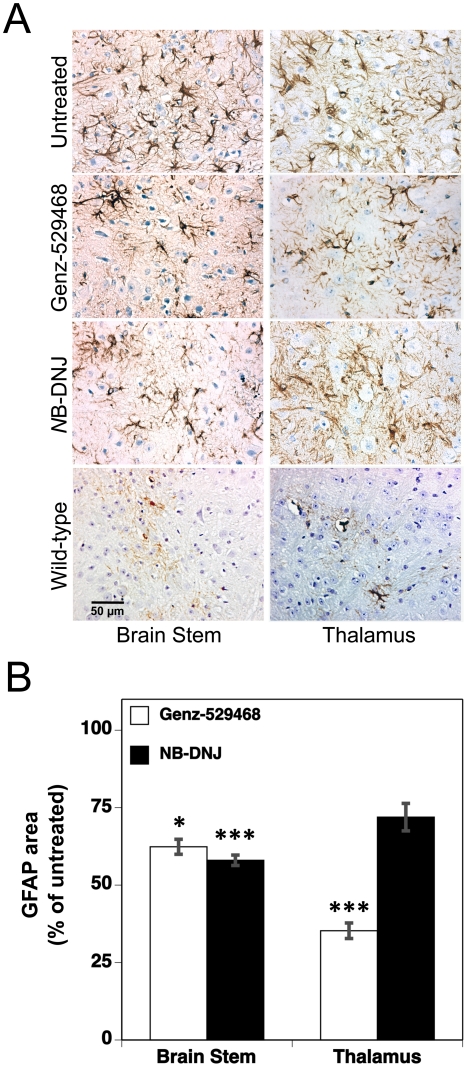
Iminosugar-based GCS inhibitors decrease brain GFAP positive cells. (**A**) GFAP immunolabeling of brain stem (left panels) or thalamus (right panels) of 112 day old wild-type or Sandhoff mice (untreated or treated with either Genz-529468 or *N*B-DNJ). Dark brown cells are positive for GFAP; scale bar = 50 µm. (**B**) Quantitative analysis of GFAP staining in brain stem and thalamus of drug-treated Sandhoff mice (n = 4–5 mice per group). GFAP stained area is presented relative to that of untreated Sandhoff mice. Statistics are between untreated and treated Sandhoff mice, and were determined using the Graphpad Prism software t test; * = p<0.05, *** = p<0.001. Error bars indicate SEM.

Glial fibrillary acidic protein (GFAP) is an astrocyte marker that increases during astrocytic activation, including in response to neurodegeneration. Elevations in GFAP staining in the brains of Sandhoff mice have been reported previously [24], [26]. In untreated Sandhoff mice, the most prominent GFAP immunolabeling was observed in the brain stem, thalamus, cerebellum, hippocampus and cerebral cortex. As with the CD68 marker, there was a temporal increase in GFAP immunolabeling within these brain regions, especially in the brain stem and thalamus. Immunohistochemical analysis of the brain stem and thalamus of Sandhoff mice treated with either Genz-529468 or *N*B-DNJ (at day 112) showed a reduction in GFAP staining when compared with age-matched wild type mice (**Figure 3A**). Quantitation of the number of GFAP-positive cells confirmed these apparent histological reductions in drug-treated animals (**Figure 3B**).

**Figure 4:**
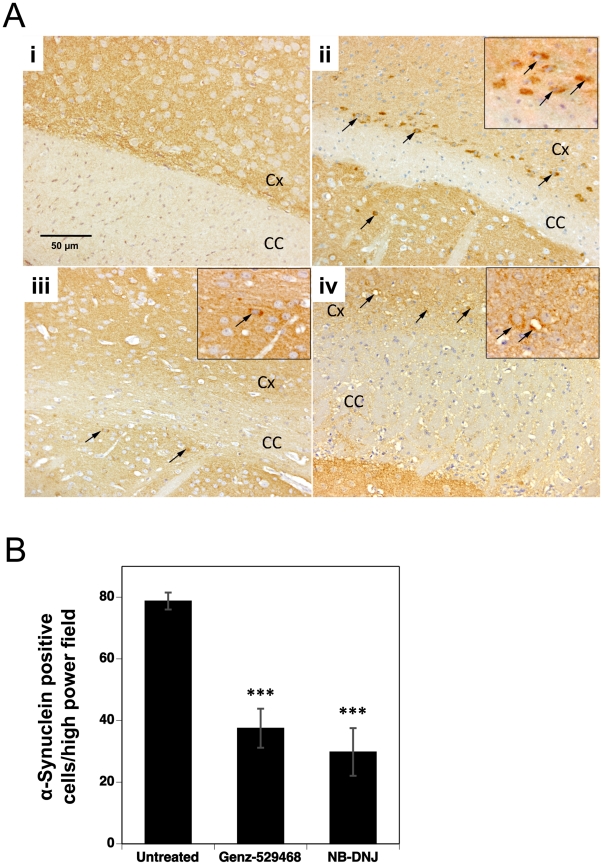
Iminosugar-based GCS inhibitors decrease brain α-synuclein aggregates. (**A**) Immunolabeling of α-synuclein in the cortex (Cx) adjacent to the corpus callosum (CC) of 112 day old (i) wild-type or (ii) untreated Sandhoff mice or (iii) Sandhoff mice treated with Genz-529468 or (iv) Sandhoff mice treated with *N*B-DNJ. Dark cells (arrows) are positive for α-synuclein; scale bar = 50 µm. Inset shows a further 3× magnification of α-synuclein positive cells. (**B**) Quantitation of α-synuclein positive cells in the cortex of untreated or drug-treated 112 day old Sandhoff mice (n = 4–5 mice per group). Statistics are between untreated and treated Sandhoff mice, and were determined using the Graphpad Prism software t test; *** = p<0.001. Error bars indicate SEM.

Accumulation of α-synuclein in the CNS is a common feature of many neurodegenerative diseases including Sandhoff disease [27]. Staining of cortical sections from 112 day-old wild type mice showed no detectable α-synuclein staining (**Figure 4A** **; panel i**). In contrast, the cortex of age-matched Sandhoff mice exhibited clear indications of positive α-synuclein staining (**Figure 4A** **; panel ii**). Similar results were seen in the hippocampus and brain stem of Sandhoff mice, with more infrequent positive staining in the striatum and thalamus (data not shown). Intense immunostaining for α-synuclein was observed in the cytoplasm of neurons in these areas. Immunoreactivity was also observed in a few cortical neurons containing cytoplasmic vacuoles (suggesting substrate accumulation). Sandhoff mice treated with Genz-529468 or *N*B-DNJ showed a reduced number of cells staining positive for α-synuclein and the intensity of staining was also lower (**Figure 4A** **; panels iii and iv**). Quantitation of the number of α-synuclein-positive cells in the cortex of 112 day-old animals showed that treatment had reduced the number by ∼50% (**Figure 4B**). Together, these data suggest that the iminosugar-based inhibitors of GCS were able to reduce the extent of inflammation and neurodegeneration in multiple brain regions of Sandhoff mice.

### Genz-529468 and *N*B-DNJ significantly delay the rate of loss of motor-coordination and locomotion in Sandhoff mice

**Figure 5:**
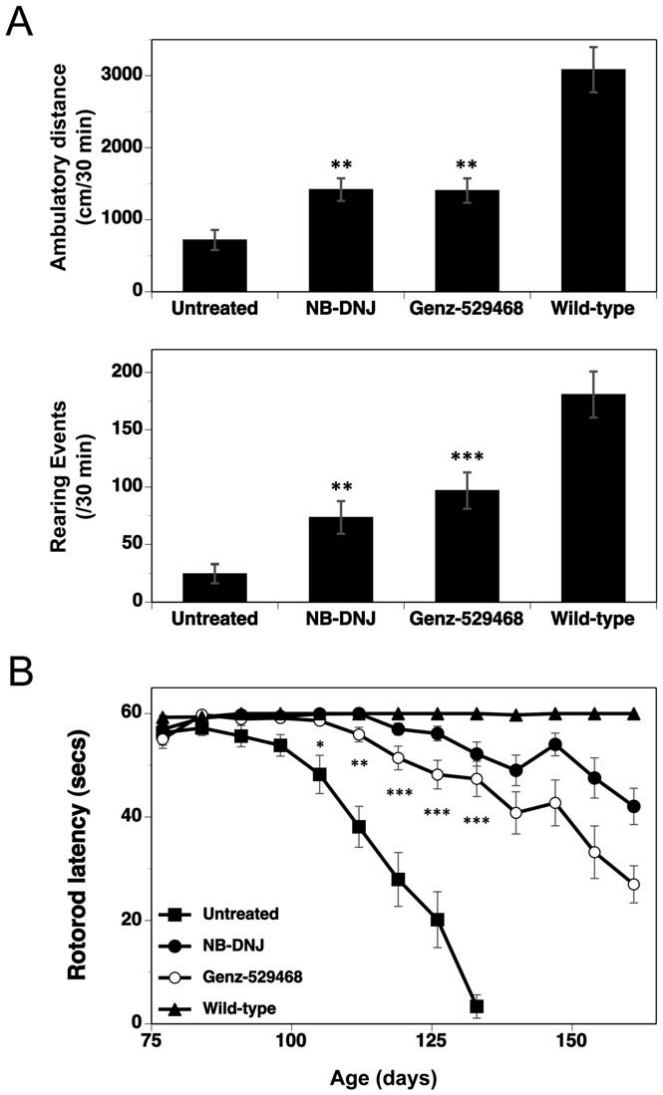
Iminosugar-based GCS inhibitors improve Sandhoff mouse function. (**A**) Mice were evaluated in an open-field assay at 112 days of age. Total distance traversed (ambulatory distance) and the number of times the mice raised onto their hind legs (rearing events) over 30 min are shown. (n = 15/group. Statistics are between untreated and treated Sandhoff mice, and were determined using the Graphpad Prism software t test; ** = p<0.01, *** = p<0.001. Error bars indicate SEM). (**B**) Mice were evaluated for motor coordination using the rotarod assay. The amount of time (in secs) the mice remained on the rotarod is reported as the latency. Latency is shown for wild-type mice, Genz-529468- and *N*B-DNJ-treated Sandhoff mice and untreated Sandhoff mice. (n = 15/group). Statistics compared untreated Sandhoff to Genz-529468-treated Sandhoff mice, and were determined using the Graphpad Prism software t test; * = p<0.05, ** = p<0.01, *** = p<0.001. Error bars indicate SEM.

Mouse locomotor ability was measured using an activity chamber apparatus (open-field) in which the mice were allowed to explore freely for 30 min. **Figure 5A** shows that at the 112 day time point, both *N*B-DNJ and Genz-529468 treated mice exhibited significantly greater ambulatory activity (as measured by the distance traversed and the number of rearing events) than the untreated Sandhoff controls. There was no significant difference between the groups treated with *N*B-DNJ and Genz-529468. These results are consistent with previous reports of Sandhoff mice treated with iminosugar-based GCS inhibitors [21], [22].

Mice were also evaluated using the elevated plus maze (habituation) and rotarod (motor coordination) assays. Motor coordination deficits have previously been demonstrated in the Sandhoff mouse model [18], [20], [26], [28]. Testing was initiated prior to the onset of overt disease symptoms and continued until either the mice were unable to perform the test, or there were fewer than 8 mice/group surviving. No measurable deficit was detected in the Sandhoff mice using the elevated plus maze test. However, untreated Sandhoff mice showed a loss of motor coordination starting at 100 days of age when tested with the rotarod assay (**Figure 5B**). Treatment with Genz-529468 significantly delayed the onset (by approximately 2 weeks) and rate of loss in motor coordination. Sandhoff mice treated with *N*B-DNJ showed a similar if not better benefit than those treated with Genz-529468 (**Figure 5B**). Age-matched wild-type mice showed no loss in motor coordination over the same period.

### Genz-529468 and *N*B-DNJ are equally effective at increasing the longevity of Sandhoff mice

**Figure 6:**
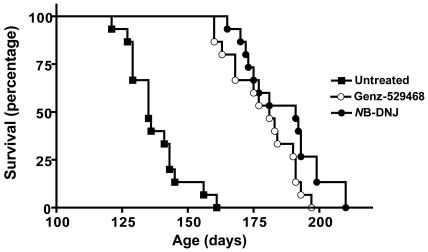
Iminosugar-based GCS inhibitors increase Sandhoff mouse survival. Mice were monitored daily from 80 days of age and euthanized when they became moribund and were unable to right themselves from a supine position within 30 sec. Untreated mice displayed a median survival of 135 days; Sandhoff mice treated with Genz-5294468 or *N*B-DNJ had median survivals of 181 days and 191 days, respectively. Both iminosugar-based GCS inhibitors significantly (p<0.0001) increased survival relative to that of untreated Sandhoff mice. (n = 15/group).

Sandhoff mice were administered the maximal effective dose of either Genz-529468 or *N*B-DNJ in their diet; for Genz-529468 this was ∼100 mg/kg/day (determined empirically) and for *N*B-DNJ ∼600 mg/kg/day [22]. A dose of 1200 mg/kg/day of *N*B-DNJ caused diarrhea and resulted in no survival benefit, although this and higher doses have previously been reported as beneficial [13], [21]. Body weights and food intake were the same between Sandhoff and wild-type mice (through day 100), and were unaffected by treatment. In agreement with previous studies [22], [28], [29], untreated Sandhoff mice became moribund (criterion for sacrifice) at a median age of 135 days (**Figure 6**). Treatment with either iminosugar-based GCS inhibitor significantly (p<0.0001) increased their lifespan; with Genz-529468, the median lifespan was 181 days and with *N*B-DNJ it was 191 days, which represented increases of 34% and 41%, respectively. There was no significant difference in survival between mice treated with *N*B-DNJ and Genz-529468. This enhancement of survival is in concordance with published reports using *N*B-DNJ [13], [21], [22], [29]. Delivery of these inhibitors across the blood-brain barrier was key to their efficacy, since Genz-112638 (eliglustat tartrate), which is non blood-brain barrier-permeant [7], [8] showed no survival benefit (median lifespan of 132 days).

## Discussion

Substrate reduction therapy has shown promise in preclinical studies as a therapeutic approach for several lysosomal storage diseases, including Gaucher [6], [7], Fabry [8], Pompe [30], and the gangliosidoses [13], [14]. However, as a tolerated dose of SRT is unlikely to completely block the synthesis of the respective storage products, this strategy is expected to be most applicable to diseases where there is some residual enzyme activity or in which an alternative mechanism or pathway exists for substrate removal. In this regard, neuropathic glycosphingolipidoses that are likely to benefit from this therapeutic paradigm are type 3 Gaucher disease, and late-onset Sandhoff and Tay-Sachs diseases [31], [32]. However, clinical trials with miglustat (*N*B-DNJ) have demonstrated limited success [33]–[36]. A suggestion was that the drug might not have sufficient potency at the tolerated doses. Consequently, we elected to evaluate the therapeutic potential of Genz-529468, another iminosugar-based inhibitor of GCS, whose IC_50_ is 250-fold greater than that of miglustat [23].

Sandhoff mice treated with either Genz-529468 or *N*B-DNJ (at their maximal effective doses) showed similar improvements in a number of parameters assayed, perhaps reflecting a shared mechanism of action for these two structurally related molecules. These improvements included a delay in the loss of motor function and coordination, reduced neuroinflammation and histopathology, as well as increased survival. Several of these observations are consistent with those reported previously for *N*B-DNJ [13], [21]. However, it would appear that the use of Genz-529468, a more potent GCS inhibitor than *N*B-DNJ, provided no additional benefit in the parameters measured, suggesting that a maximal therapeutic effect might have been attained with this class of GCS inhibitors.

Profiling the glycosphingolipids in the livers of Sandhoff mice treated with the GCS inhibitors revealed the expected lowering of the levels of the glycosphingolipids GL1, GM2 and GA2. Paradoxically, analysis of brain lipids from treated Sandhoff mice showed a dramatic increase in the GL1 levels as well as significant increases in other glycosphingolipids. This finding of higher lipid levels (GM2) in the brains of drug-treated Sandhoff mice had been reported previously [22] and also for another iminosugar-based GCS inhibitor, *N*B-DGJ [21], though these were both end-stage measurements. The basis for the observed increase in brain GL1 levels by these iminosugar-based GCS inhibitors was likely due to its reported secondary inhibitory activity of the non-lysosomal enzyme β-glucosidase 2 (*Gba2*) [16], [37], [38]. *Gba2* is a plasma membrane-associated enzyme involved in GL1 homeostasis and is expressed maximally in testis and brain tissue [37]. Consistent with this suggestion is the observation that *Gba2* knockout mice develop elevated levels of GL1 in the brain, though with no apparent detrimental effects on health [37].

GL1 accumulation has also been previously reported in the testis and brain tissue of wild-type mice treated with this class of GCS inhibitors [39]. This increase in GL1 levels probably led to the observed increased levels of the additional complex glycosphingolipids, presumably through greater synthesis. Previous studies using *N*B-DNJ in the Sandhoff mouse had not reported altered brain GL1 levels [13], [21], [22], [40], possibly because some assay methods do not easily differentiate galactosylceramide from glucosylceramide, and galactosylceramide is generally present in a 10–20 fold excess over GL1 in the mouse CNS. These data suggest that the survival benefit elicited by the iminosugar-based GCS inhibitors might not be primarily due to substrate reduction in the CNS. It is possible that the increase in survival reflected a delay in the onset or severity of disease manifestations in the visceral organs. Indeed, bone marrow transplantation of Sandhoff mice [28] has been shown to reduce storage pathology in the visceral organs but not the brain but nevertheless conferred a 3 month extension in longevity [28]. However, as the non-CNS permeant GCS inhibitor (Genz-112638) did not provide the same improvements noted with the CNS-permeant inhibitors (Genz-529468 and *N*B-DNJ), this could not be the sole explanation.

The documented pathophysiology of neuropathic diseases such as Sandhoff [41] and the complex roles of gangliosides in the CNS [24] provide some potential mechanisms of action through which the iminosugar-based GCS inhibitors might have worked to effect the observed positive outcomes. For example, it is possible that their activities altered the extent of neurodegeneration, inflammation, autophagy and intracellular calcium regulation. Changing the lipid profiles in the brain to contain higher levels of GM1 and GL1 and lower levels of sphingosine-1-phosphate could have contributed to moderating disease severity. GM1 has been shown to enhance the functional recovery of damaged neurons [42], and GL1 reportedly can stimulate neuronal growth and development [43]. The noted Genz-529468-mediated reduction in sphingosine-1-phosphate levels could also have translated to a reduction in astroglial proliferation in the Sandhoff mice as suggested previously [44]. As inflammation is a major pathophysiologic feature of Sandhoff disease [24], [45] and a contributor to neurodegeneration or apoptosis [46], these inhibitors could also be acting to limit the inflammatory response. Anti-inflammatory drugs have been reported to provide a survival benefit in the Sandhoff mouse [26], [29]. Similarly, survival benefit following bone-marrow transplantation in Sandhoff mice has been postulated as being through an anti-inflammatory mechanism [22], [28]. Genz-529468 exhibits systemic anti-inflammatory properties [47], [48], which raises the possibility that this might be part of the basis for the improved survival seen in the treated Sandhoff mice. Brains of animals treated with Genz-529468 showed less astrogliosis and microglial activation, which in turn might have reduced the degree of neuronal damage. Treatment also caused significant reductions in both the intensity and number of α-synuclein positive aggregates in the brain. In murine models of Parkinson's disease, aggregates of α-synuclein have been shown to activate microglia and amplify neurodegenerative processes [49], [50].

In summary, these studies clearly demonstrated and confirmed the ability of iminosugar-based GCS inhibitors to delay the onset of disease and increase the longevity of a mouse model of Sandhoff disease. However, contrary to prior suggestions [13], [21], [22] it would appear that these benefits are unrelated to substrate reduction therapy, since treatment led to elevated levels of glycosphingolipids in the brain. Potential alternate mechanisms to explain the observed benefits of this class of drugs might be through their ability to (i) lessen the extent of α-synuclein aggregation, (ii) act as an anti-inflammatory agent or (iii) inhibit the non-lysosomal β-glucosidase resulting in altered levels of neuronal glycosphingolipids. Further studies are necessary to elucidate fully the basis for the neurologic benefits of this class of GCS inhibitors in Sandhoff mice.

## Materials and Methods

### Animal studies

Ethics Statement: Procedures involving mice were reviewed and approved by Genzyme Corporation's Institutional Animal Care and Use Committee (Protocol 07-1115-2-BC) following guidelines established by the Association for Assessment of Accreditation of Laboratory Animal Care. The review board specifically approved all the studies (identification numbers 09-3706, 09-3784, 09-4157, 09-4231) reported in this manuscript. Sandhoff mice [18] were purchased from Jackson Labs (Bar Harbor, ME) and contract bred at Charles River Labs (Bedford, MA). This mouse model develops neurodegenerative disease and exhibits physical difficulties in feeding, drinking and grooming at about 100 days of age. To minimize the potential for suffering, mice were assessed daily from day 80 and euthanized when they were unable to right themselves from a supine position within 30 sec.

### Drug dosing

Beginning at 3–4 weeks of age, animals were given drug as a component of the pellet food diet. Drugs were formulated at 0.05% (Genz-529468) or 0.3% (*N*B-DNJ) w/w in a standard mouse chow (LabDiet 5053, TestDiet, Richmond, IN) and provided *ad libitum*. This formulation was calculated to provide 100 mg/kg of Genz-529468 or 600 mg/kg of *N*B-DNJ per day for a 25 g mouse eating 5 g of food per day. These doses of the GCS inhibitors were considered maximal based on pilot tolerability and efficacy studies.

### Functional studies

Mice were evaluated for motor coordination and locomotion using accelerating rotarod and open-field assays, respectively. Tests were run weekly at the same time of day on each occasion. The rotarod assay consisted of placing the animals on a 30 mm diameter spindle at a height of 30 cm. The Smartrod program (AccuScan Instruments, Columbus, OH) controlled the acceleration from 0–15 rpm over 60 s. The time to fall (latency) was automatically recorded by a light beam sensor underneath the spindle. Each animal was subjected to 4 trials at each time point (the first result on each assay day was discarded), with an ∼30 min rest period between each trial. The open-field assay (Med Associates, Georgia, VT) was used to measure locomotor activity. Mice were placed individually into a 30 cm/side square high-walled arena. Movement was automatically detected using a series of sensor light beams to measure horizontal and vertical movement. Trials were performed for 30 min in a noise-controlled room. Data were analyzed using Activity Monitor software (Med Associates, St. Albans, VT) to evaluate total ambulatory movement and rearing.

### Tissue processing and immunohistochemistry

Mice at 28, 56, 84 and 112 days of age were transcardially perfused with 0.9% sodium chloride solution and tissues fixed in fresh 4% paraformaldehyde for 2 days at 4°C. Tissues were embedded in paraffin and 5 µm sections were cut. CD68 staining was performed using the Bond Polymer Refine Detection System (BPRDS; Leica Microsystems, Bannockburn, IL). Brain sections were incubated with Proteinase K (DAKO, Carpinteria, CA) for 5 min for antigen retrieval prior to staining. Sections were then incubated with either an anti-CD68 antibody (clone FA-11; Serotec, Raleigh, NC) or an isotype-matched non-specific antibody (rat IgG_2a_; Serotec, Raleigh, NC). Secondary detection was with a rabbit anti-rat antibody (Vector Labs, Burlingame, CA). Glial Fibrillary Acidic Protein (GFAP) staining was performed using the BPRDS and an anti-GFAP antibody (DAKO, Carpinteria, CA). An isotype-matched, non-specific antibody (rabbit IgG, Jackson Immunoresearch, West Grove, PA) was used as the negative control. For α-synuclein staining, antigen retrieval was achieved by treatment with 70% formic acid (Sigma, St. Louis, MO) for 10 min prior to boiling in Antigen Unmasking Solution (Vector Labs, Burlingame, CA) for 30 min. Endogenous peroxidase activity was quenched by immersion in 0.3% hydrogen peroxide (Sigma) in methanol for 30 min and α-synuclein was detected by incubating with rabbit anti-α-synuclein antibody (Sigma) overnight at 4°C. Visualization was achieved using goat anti-rabbit-HRP followed by diaminobenzidine (DAB) and counterstained with hematoxylin.

For quantitation, three non-overlapping fields of view for each brain region were examined at 400× magnification (for counting CD68 positive cells) or 200× (for counting α-synuclein positive cells) with an n of at least 3 per group. GFAP immunopositive areas were quantitated at 400× in the brain stem and thalamus using the MetaMorph image analysis software (Molecular Devices, Inc., Sunnyvale, CA). The images were thresholded for brown areas corresponding to GFAP immunostaining. GFAP immunopositive area in each section was determined using three representative, non-overlapping images.

### Sphingolipid analysis

Quantitative analysis of sphingolipids was performed by liquid chromatography and tandem mass spectrometry (LC/MS/MS) [51]. Briefly, tissue was homogenized in 10 times its volume of water (w/v) and 10 µl of homogenate was extracted with 1 ml of an organic solvent mixture (acetonitrile, methanol, acetic acid, 50/50/1∶v/v/v) for 10 min under strong vortex. For sphingomyelin, phosphatidylcholine, GL2, GM3, GM2, GA2 and GM1 analyses, an Acquity HILIC column (2.1×100 mm, Waters Corp., Milford, MA) was used to separate the glycosphingolipids and phospholipids that were then analyzed by triple quadrupole tandem mass spectrometry (API 4000, Applied Biosystems/MDS SCIEX, Carlsbad, CA) using MRM mode. An Atlantis HILIC column (Waters Corp., Milford, MA) was used to separate GL1 and galactosylceramide prior to detection by tandem mass spectrometry (API 4000). For ceramide and sphingosine-1-phosphate analyses, a reverse phase column (Acquity C8 2.1×100 mm, Waters Corp., Milford, MA) was used to separate different isoforms of ceramide before analysis by tandem mass spectrometry (API 5000 detector). Sphingolipid standards were obtained from Matreya, LLC (Pleasant Gap, PA).
